# Charting Availability of Processed and Unprocessed Foods in School Neighbourhood Nutrition Environments in an Urban Australian Setting

**DOI:** 10.1155/2017/8397469

**Published:** 2017-05-03

**Authors:** Holly Oaken, Lisa Vaughan, Nicola Fa'avale, Robert S. Ware, Lisa Schubert

**Affiliations:** School of Public Health, Faculty of Medicine, The University of Queensland, Public Health Building, Herston, QLD 4006, Australia

## Abstract

School Neighbourhood Nutrition Environments (SNNEs) can facilitate or impede healthy eating. This study describes the SNNEs surrounding 6 Good Start Program (GSP) schools in 5 suburbs in Logan, Queensland. Relative density of healthy and unhealthy food outlets was calculated for SNNEs surrounding GSP (6) and non-GSP (10) schools within the 5 suburbs. Relative accessibility of minimally processed and highly processed food and drink in SNNEs of the 6 GSP schools was determined using shelf measurements of snack foods. Unhealthy outlets greatly outnumber healthy outlets (mean relative density 15.6%, median 19.1%). The majority of outlets stock predominantly highly processed food and drink. Study areas are dominated by unhealthy food outlets and highly processed food.

## 1. Introduction

Globally, the prevalence of overweight and obesity has been increasing for both adults and children, representing a significant public health issue. There are a range of factors contributing to the increasing rates with poor diet being a major contributing factor. The Australian National Survey (2011-12) has shown that the majority of Australians do not meet the minimum recommended serves for the five major food groups. Among children, less than 1% are estimated to meet their recommended number of serves of vegetables and legumes/beans on a usual basis [1]. Fruit consumption is considerably better; however it decreases with age with 78% aged 2-3 years, 59% aged 4–8 years, 39% 9–13 years, and 27% 14–18 years meeting the recommendations [1]. A further finding of the survey was that “over one-third of the population's total daily energy intake came from energy-dense, nutrient-poor ‘discretionary foods' (such as sweetened beverages, alcohol, cakes, confectionary, and pastry products)”. Additionally, surveys show that a quarter of school-aged Australian children consume fast food at least once per week, rising to 43% during adolescence [2].

The reasons behind food choices are complex, with the food environment being one of the potential contributors, particularly the food retail environment and the increasing availability and accessibility of fast food and convenience stores [3]. In industrialised countries, those with the greatest risk of poor dietary behaviours include children and families from ethnic minorities and low income groups exposed to poor food environments; both high concentrations of fast food and convenience stores and a scarcity of supermarkets are key features of these environments [3–5]. Additionally, the marketing of fast-foods, which makes them highly attractive to children and adolescents, has been shown to be another significant influence on food choices [6, 7], leading to the growing interest in the regulation of fast food advertising [8, 9].

Community nutrition environments and consumer food environments are increasingly being investigated internationally as potentially important contributing factors shaping dietary behaviours [10], including for children. In this paper we have used the concept of School Neighbourhood Nutrition Environments (SNNEs), to explore these two environmental variables in the school vicinity. Absolute density has been used to document the clustering of unhealthy outlets around schools [11–14], the association between unhealthy food outlets near schools and students' BMI [15–17], and the association between unhealthy food outlets and eating and/or purchasing behaviours [15, 18–21]. To date, findings are mixed and any associations appear to be complex [18]. Some studies have found no statistically significant association between the school neighbourhood environment and student eating behaviour [22, 23] or BMI [24, 25].

Australian research suggests that the relative density of healthy to unhealthy outlets may be more predictive of healthy and unhealthy purchasing by adult participants than the absolute density of outlets [26]. One Australian study on children showed that the likelihood of fruit consumption decreased when fast food outlets and convenience stores were closer to home, and vegetable consumption increased the farther the children lived from a fast food outlet or supermarket [27]. However, access to fast food was not predictive of fast food consumption [28]. These relationships appear complex and may not be sufficiently captured by the measures used. Within Logan, Queensland, the community nutrition environment has been identified as a notable influence on dietary behaviour [29].

Relative accessibility of food items, such as comparing shelf measurements of processed snack foods to fruit and vegetables (FV), is a method used to measure consumer nutrition environments [8, 30–33]. Relative accessibility has also been shown to have an effect on the purchase of different products [30, 34].

To date, there has been no research focused specifically on describing SNNEs in Australia. The aim of this study was to describe the SNNE surrounding six schools in a low socioeconomic area of Logan, Australia. The relative density of healthy and unhealthy food outlets was determined, as was the relative accessibility of minimally processed and highly processed foods in all outlets within 1 km of each selected school.

## 2. Methodology

### 2.1. Study Design

This cross-sectional study was conducted in Logan, Australia, between October 2014 and May 2015. The present study was initiated to complement ongoing Good Start Program (GSP) evaluation. The GSP is a health intervention that aims to improve the health and wellbeing of Maori and Pacific Island (MPI) children and their families [35]. Logan City has one of the highest concentrations of MPI populations of anywhere in Australia with 25% of migrants arriving in the five years prior to 2003 [36]. Logan City has some of the highest areas of relative socioeconomic deprivation in Australia, as measured by Socioeconomic Indexes for Areas (SEIFA) [37]. In the areas chosen for this study, only 4–19% of Australian areas had higher levels of deprivation [36]. Six schools, four primary and two secondary, in which the Good Start Program was operating were selected: the GSP SNNEs.

### 2.2. Food Outlet Data for Relative Density

The first study objective was to determine the relative density of healthy and unhealthy outlets in the GSP SNNEs together with an additional 10 schools that were within these suburbs. The SNNE was defined as a 1 km radius around each of the study schools, as several studies have shown that 1 km radius is typically “walking distance” [16, 19, 38]. School location data was retrieved from the Queensland Government online schools directory [39], geocoded, and entered into ArcGIS (2016) [40]. Ethical approval was not sought, as the research did not involve any human subjects.

A register was compiled of all known food outlet businesses in the Logan municipality from the Logan Municipal Council (LMC) records, as it has been previously determined that government records represented the most accurate records [41]. Online business records were used to complete the register, before nonrelevant businesses and duplicates were removed. Outlets removed included places children were unlikely to visit, such as sit-down restaurants and licensed premises. Addresses were geocoded and entered into ArcGIS Map.

Outlets were organized into four categories: takeaway/fast food outlets, corner stores, green grocers, and grocery stores. Takeaway/fast food outlets were considered any business whose primary service was the provision of ready-to-eat or quick-service meals. Corner stores were a broad category including independently owned convenience stores and petrol stations. Takeaway/fast food outlets and corner stores were categorized as unhealthy outlets [26]. Grocery stores and green grocers were categorized as healthy outlets because they are the primary sites of fruit and vegetable purchasing [26]. Ground-truthing was performed for all outlets within the SNNEs to verify the businesses were as described in the LMC register and online, as well as checking for additional premises, by physically viewing each of the locations.

### 2.3. Store Inventory Data for Relative Accessibility

#### 2.3.1. Outlet and Inventory Inclusion Criteria

The second study objective was to determine the relative accessibility of minimally processed and highly processed food and drink. All known outlets selling snack foods and drinks within the six GSP SNNEs were included. The lead researcher (HO) visited each of the outlets identified. Permission was sought from staff. Some outlets declined as they were parts of larger chains and required approval from head office, or because a manager was not present.

Items accessible to students were identified, defined as food and drink that a school-aged child could plausibly purchase and consume with minimal preparation [42]. Items were divided into four broad categories: unprocessed/minimally processed/basic processed food, moderately/highly processed food, unprocessed/minimally processed/basic processed beverages, and moderately/highly processed beverages using the category definitions and criteria used for classifying foods and beverages developed by Poti et al. [43], summarized in Table 1. Existing validated methods available for collecting local level store inventory data focus on accessibility of healthy options, or relative accessibility of a limited range of food categories [10], rather than auditing all food and beverage categories available, and therefore did not fit the purpose of the present study. Unprocessed/minimally processed/basic processed food will be hereafter referred to as minimally processed food, and moderately/highly processed food will be referred to as highly processed food.

#### 2.3.2. Measurement of Store Inventory

For each store visited, the linear distance in metres for shelving space was recorded and summed for all shelving space displaying items meeting the inclusion criteria. Each portion of the shelf was measured along the front base of the shelf where products were visible. Display “islands” in the centre of aisles were measured from all sides [30, 33]. Cold drink fridges were treated as other shelves, with each shelf holding bottles measured individually. Measurement did not take into consideration the height or depth of the shelves.

The availability of fresh FV anywhere in the store was recorded, and whether any impulse minimally processed food items were available within arm's length of the cash register [44].

### 2.4. Presentation of Data

Relative density was expressed as the percentage of healthy outlets out of the total number of outlets within a given area. In addition to the six GSP study schools identified, there were a further 10 schools within these suburbs. Relative availability of healthy and unhealthy outlets was calculated for 1 km buffer zones surrounding each of the 16 schools in order to provide further context on the overall makeup of the identified suburbs. The mean, standard deviation, range, median, and quartiles were calculated for the number of outlets. The percentage of healthy outlets out of the total number of outlets was also calculated.

## 3. Results

### 3.1. Relative Availability of Healthy and Unhealthy Outlets

The final register of outlets meeting inclusion criteria in the municipality of Logan included 217 takeaway/fast food outlets, 83 supermarkets/grocery stores/green grocers, and 71 convenience stores/corner stores/petrol stations. Outlets excluded from the study totalled 151, as they were cafes, bakeries, and confectionary/ice cream shops which did not fit the study's definition of healthy/unhealthy stores. The total number of outlets within individual SNNEs is shown in Supplementary Table 1 (see Supplementary Material available online at https://doi.org/10.1155/2017/8397469).

Table 2 shows the summary statistics of the number of outlets identified in the 1 km circular buffers surrounding each school in the study area, as well as the percentage of healthy outlets.

### 3.2. Snack Food Outlet Identification

Fifteen outlets in the grocery store/convenience store categories were initially identified within the six 1 km buffer zones of the GSP schools. Ground-truthing brought the total number to 22. Five (23%) outlets declined to participate in the study. In total, an inventory was taken of 17 food outlets, including 9 corner stores, 6 grocery stores, and 2 green grocers. These outlets were all included in the shelf space analysis.

### 3.3. Relative Accessibility of Minimally Processed and Highly Processed Items

Table 3 shows a summary of the shelf space data gathered on each of the outlets visited (*N* = 17).  Table 4 shows descriptive statistics for the shelf space devoted to highly processed items as a percentage (%) of total items.

The overall distribution favoured highly processed items. The average percentage of shelf space occupied by highly processed food items was 88%, and the median percentage was 96%. The only notable outliers were in the case of the two green grocers surveyed, where 28.1% and 33.6% of snack food product shelving space were used to stock highly processed foods. 29% (*N* = 5) of outlets surveyed sold exclusively highly processed food products. The most common items observed were salty snacks, confectionary, and single-serve ice creams.

Overall, 41.2% (*N* = 7) of outlets sampled sold any fresh FV. All of the outlets stocking fresh FV were supermarkets or green grocers. None of the corner stores visited stocked fresh FV. 11.7% (*N* = 2) of outlets surveyed stocked at least one minimally processed food item in the impulse buy area. In both cases, the minimally processed food near the cash register was bananas.

Table 5 details the total amount of shelving space displaying all snack-size drinks, the total space displaying highly processed snack-sized drinks, and the percentage of space devoted to highly processed drinks.

As with food, the ratio of shelf space favoured processed drinks. An average of 86% of drink shelf space was used to stock highly processed drinks. The most common highly processed drink items observed were soft drinks, juice drinks, energy drinks, and iced coffee beverages. The most common minimally processed drink items observed were water, unflavoured milk and milk alternatives, and unsweetened juice. One outlet visited stocked no minimally processed drinks.

## 4. Discussion

The results of this study indicate there is a relative abundance of unhealthy food available in school neighbourhoods in Logan, Queensland, an area of high socioeconomic deprivation with a high migrant population. Healthy outlets were found to make up less than 25% of the total number of outlets in each of the 16 SNNEs. These results were similar to previous research in other areas of Australia [26]. Additionally, highly processed foods and beverages were found to take up a large percentage of total shelving space in the SNNEs surrounding the identified GSP schools, even in stores where healthy food was available. No corner stores surveyed stocked fresh fruit or vegetables. This observation that the distribution of shelf space is largely skewed towards processed snack foods is also in line with previous research on this topic [30–32].

The strengths of this study were that a variety of methods were used to describe the SNNEs. Previous studies on SNNEs have frequently focused on absolute density, which does not provide a clear picture of the mix of outlets and products available in an area. This study instead looked at relative density, which may be more closely linked to FV consumption [26]. Also, while the relative availability of minimally processed and highly processed products has been used to represent the distribution of products that a consumer will encounter in stores in a given area, to our knowledge, this strategy has not yet been applied to school neighbourhoods.

There are also some limitations to the study. First, as this study focused exclusively on 5 Logan suburbs, these results are not generalizable; however, the methodology is transferable to other sites. Second, despite thorough efforts to collect exhaustive data, it is likely that some outlets were missed. Due to time constraints, ground-truthing was only carried out in the 6 GSP SNNEs rather than in all 16 SNNEs in the study area. Furthermore, several stores declined to participate and were not included in the analysis. Third, while circular buffers were used to determine the 1 km radius defining SNNEs in line with similar research studies, road network buffers that are created using an area's road network are thought to more accurately represent* where* a person would walk [45]. Fourth, several other factors relevant to purchasing behaviour fell outside of the scope of this study, including the range, cost, and quality of items available, as well as which outlets are being patronized, and purchasing motivations. And finally, availability of items are also subject to change.

Despite these limitations, the localized data collected as part of this study provides useful information for the Logan GSP schools, and public health response planning in the Logan area. While programs like the GSP strive to encourage healthy eating habits among students, the neighbourhoods in which they exist provide relatively limited opportunities to make those healthy choices. At the local school level, information regarding the SNNE around each school could be incorporated into the GSP program or other healthy eating initiatives. This could involve practical, local advice for navigating an environment where healthy food options may be difficult to locate.

This research also highlights research strategies that could be useful in the context of other childhood obesity prevention interventions. One of the primary strengths of the GSP was the centrality of the multicultural health workers in developing and administering the program [46]. Feedback from the multicultural health workers and community stakeholders on the program was essential in ensuring that the program adequately addressed the needs of the community [46]. The development of the current study in response to observations made by key stakeholders recognises the importance of community participation in setting research agendas that could support place-sensitive initiatives targeting childhood obesity.

The areas surveyed in Logan, where we found an absence of fresh FV in corner stores, may benefit from creative solutions already in practice internationally. The Change4Life program in the UK has used partnerships with local businesses, in areas of high deprivation, to improve the availability of healthy food options in small food outlets. Outlets involved in the more intensive intervention saw between a 6% and 480% increase in FV sales after 6 months (average increase 143%) [47]. The outlets involved in the less intensive intervention saw lower, but consistent, increases in FV sales, with an average 13.5% increase. [47]. The program was popular with participating retailers and customers [47]. Despite the fidelity and processes of this particular intervention implementation being critiqued [48], the identified weaknesses were not insurmountable. Rather, they would need to be taken into consideration as “lessons learned” with any future intervention planning.

Local research has found that residents of low socioeconomic status neighbourhoods in Australia are concerned about the number of fast food outlets in their area and found this environment to be a barrier to healthy eating [49]. Some local governments in Europe have sought to alter SNNEs by banning sales of “junk food” near schools in Germany [50] and restricting the opening of new takeaway outlets in London [51]. Municipalities in Queensland may be in a better position to argue for public planning approaches, as, unlike other states, health considerations are included in the state's principal legislation for sustainable planning [52].

Further research is needed on both primary and secondary students and local businesses to help inform the direction of local public health initiatives. In particular, qualitative and observational research will be valuable in the following areas: current purchasing habits and decision-making processes of students, and barriers to stocking healthier products in local businesses [see [53, 54] for examples of relevant Canadian research].

## 5. Conclusion

The School Neighbourhood Nutrition Environments in the Good Start Program suburbs in Logan, Australia, are characterized by an abundance of unhealthy outlets relative to healthy outlets. Individual stores in the GSP SNNEs primarily stock highly processed food and drink. None of the corner stores surveyed stocked any fresh fruit or vegetables. This environment may be currently undermining local initiatives to encourage healthy eating habits, such as the Good Start Program. International examples exist of partnerships with local businesses and municipal zoning changes that can positively alter the local nutrition environment. However, further research is required to adapt and implement successful initiatives to this local context.

## Supplementary Material

Supplementary Table 1: Total number of outlets within individual School Neighbourhood Nutrition Environments within 1 km buffer zone of each school.

## Figures and Tables

**Table 1:** Summary of degrees of processing of food and drinks.

Category	Definition	Example
Unprocessed/minimally processed food items	Single-ingredient foods with no or very slight modifications	Plain milk, fresh, frozen, or dried fruit and vegetables, and plain nuts

Basic processed food items	Single food components “obtained by extraction or purification” or “processed for basic preservation or precooking” [43, p. 1253]	Unsweetened fruit juice and canned fruit or vegetables

Moderately processed food items	Single minimally or moderately processed foods with addition of flavour additives for the purpose of enhancing flavour	Sweetened/flavoured juice or milk, potato chips, whole-grain bread products, and sweetened/flavoured yogurt

Highly processed food items	Multi-ingredient industrially formulated mixtures processed to the extent that they are no longer recognizable as their original plant/animal source	Fruit drinks, sports drinks, soft drinks, energy drinks, coffee beverages, flavoured water, fruit snacks, processed lunchmeats, refined-grain bread products, cookies, pastries, salty snacks, ice cream, candy, chocolate, and popsicles

**Table 2:** Summary statistics of numbers of outlets within School Neighbourhood Nutrition Environments (within 1 km buffer zone of each school) overall, grouped by school type.

	SNNEs (*n*)	Mean	SD	Range	25th percentile	Median	75th percentile	% of healthy outlets
*Overall*								
All SNNEs	16	12	±10.2	1–35	5	10	18	22%
*By school type*								
Primary school SNNEs	9	13.7	±11.8	1–35	6	11	27	19.4%
Secondary school SNNEs	5	8.4	±7.6	1–18	3.5	5	17.3	11.9%
K-12 SNNEs	2	13	±11.3	5–21	*∗*	13	*∗*	23.1%

^*∗*^Percentiles not calculated as only two data points in category.

**Table 3:** Store shelf space devoted to minimally processed and highly processed snack foods in outlets (*N* = 6) within 1 km of selected primary schools (*N* = 4) and outlets (*N* = 11) within 1 km of selected secondary schools (*N* = 2).

Outlet	Outlet type	Total food shelving (m)	Total highly processed food (m)	Highly processed food of total food (%)	FV available	Minimally processed impulse food available
*Primary schools*						
1	Corner store	50.0 m	50 m	100%	No	No
2	Grocery store	83.0 m	81.9 m	98.6%	Yes	No
3	Green grocer	39.0 m	13.0 m	33.3%	Yes	Yes
4	Grocery store	29.2 m	29.2 m	100%	No	No
5	Grocery store	104.1	101.7 m	97.7%	Yes	No
6	Grocery store	92.5 m	85.1 m	92%	Yes	No
*Secondary schools*						
7	Grocery store	27.2 m	25.8 m	94.9%	Yes	Yes
8	Green grocer	19.4 m	5.5 m	28.1%	Yes	No
9	Corner store	48.7 m	46.2 m	94.8%	No	No
10	Corner store	22.9 m	20.7 m	90.2%	No	No
11	Grocery store	65.1 m	55.5 m	85.3%	Yes	No
12	Corner store	5.0 m	5.0 m	100%	No	No
13	Corner store	4.6 m	4.6 m	100%	No	No
14	Corner store	29.0 m	29.0 m	100%	No	No
15	Corner store	61.6 m	55.4 m	89.9%	No	No
16	Corner store	77.5 m	74.0 m	95.6%	No	No
17	Corner store	54.9 m	53.4 m	97.3%	No	No

**Table 4:** Summary statistics of shelf space devoted to highly processed items as a percentage (%) of total items.

	Mean	SD	Range	25th percentile	Median	75th percentile
Total food	88	±22	72	91	96	1
Total drink	86	±11	34	83	91	95
Food in primary school SNNEs	87	±26	67	95	98	1
Food in secondary school SNNEs	89	±21	72	90	95	1
Drink in primary school SNNEs	82	±13	27	75	87	94
Drink in secondary school SNNEs	89	±9	32	86	92	96

**Table 5:** Store shelf space devoted to minimally processed and highly processed drinks in outlets (*N* = 6) within 1 km of Good Start Program primary schools (*N* = 4) and outlets (*N* = 11) within 1 km of Good Start Program secondary schools (*N* = 2).

Outlet	Outlet type	Total drink shelving (m)	Total highly processed drink (m)	Highly processed drink of total drink (%)
*Primary schools*				
1	Corner store	27.20 m	22.49 m	82.35%
2	Supermarket	42.29 m	39.5 m	93.39%
3	Greengrocer	18.03 m	11.94 m	66.2%
4	Supermarket	20.14 m	13.54 m	67.21%
5	Supermarket	50.57 m	46.1 m	91.16%
6	Supermarket	36.86 m	34.53 m	93.69%
*Secondary schools*				
7	Supermarket	26.67 m	23.62 m	88.57%
8	Greengrocer	12.7 m	12.07 m	95%
9	Corner store	20.02 m	18.36 m	91.75%
10	Corner store	4.83 m	3.96 m	82.1%
11	Supermarket	26.87 m	25.59 m	95.23%
12	Corner store	4.8 m	4.53 m	94.44%
13	Corner store	3.45 m	3.45 m	100%
14	Corner store	15.63 m	10.68 m	68.32%
15	Corner store	36.93 m	31.85 m	86.24%
16	Corner store	31.72 m	26.7 m	84.19%
17	Supermarket	43.18 m	42.42 m	98.25%
