# Supplementary material for: Charting Availability of Processed and Unprocessed Foods in School Neighbourhood Nutrition Environments in an Urban Australian Setting
Source: J Environ Public Health. 2017 May 3;2017:8397469. doi: 10.1155/2017/8397469 (PMC5434266; doi:10.1155/2017/8397469)
Supplement: Supplementary file 1 — Supplementary Table 1: Total number of outlets within individual School Neighbourhood Nutrition Environments within 1 km buffer zone of each school. [file 8397469.f1.docx]

**Supplementary Tables**

Supplementary Table 1 below shows the outlet data collected on the SNNEs surrounding each of the 16 schools in the 5 study suburbs in Logan. Schools participating in the GSP are italicized.

**Supplementary Table 1.**

Total number of outlets within individual School Neighbourhood Nutrition Environments (within 1km buffer zone of each school)

|  | School type | Unhealthy outlets | Healthy outlets | Total outlets | % of healthy outlets |
| --- | --- | --- | --- | --- | --- |
| **Woodridge** |  |  |  |  |  |
| Woodridge North State School | Primary | 7 | 2 | 9 | 22.2 |
| *Harris Fields State School* | Primary | 14 | 4 | 18 | 22.2 |
| St. Paul’s School Woodridge | Primary | 28 | 7 | 35 | 20 |
| **Kingston** |  |  |  |  |  |
| YMCA Vocational School | Secondary | 4 | 1 | 5 | 20 |
| *Kingston State School* | Primary | 4 | 1 | 5 | 20 |
| Groves Christian College | K-12 | 4 | 1 | 5 | 20 |
| Kingston College | Secondary | 3 | 0 | 3 | 0 |
| *Berrinba Primary School* | Primary | 3 | 1 | 4 | 25 |
| **Marsden** |  |  |  |  |  |
| Centre Education Programme | Secondary | 1 | 0 | 1 | 0 |
| Burrowes State School | Primary | 9 | 2 | 11 | 18.2 |
| *Marsden State School* | Primary | 1 | 0 | 1 | 0 |
| **Waterford West** |  |  |  |  |  |
| *Marsden State High School* | Secondary | 14 | 1 | 15 | 6.6 |
| Waterford West State School | Primary | 9 | 2 | 11 | 18.2 |
| **Logan Central** |  |  |  |  |  |
| *Woodridge State High School* | Secondary | 15 | 3 | 18 | 16.7 |
| Logan City Special School | K-12 | 16 | 5 | 21 | 23.8 |
| Woodridge State School | Primary | 25 | 5 | 30 | 16.7 |
